# Supplementary material for: Ice-VII-like molecular structure of ambient water nanomeniscus
Source: Nat Commun. 2019 Jan 17;10:286. doi: 10.1038/s41467-019-08292-0 (PMC6336866; doi:10.1038/s41467-019-08292-0)
Supplement: Supplementary file 1 — Supplementary Information [file 41467_2019_8292_MOESM1_ESM.pdf]

Supplementary information

"Ice-VII-like Molecular Structure of Ambient Water Nanomeniscus"

Center for 0D Nanofluidics, Institute of Applied Physics, Department of Physics and Astronomy, Seoul National University, Seoul 08826, Republic of Korea

1. Contribution of added bulk water.

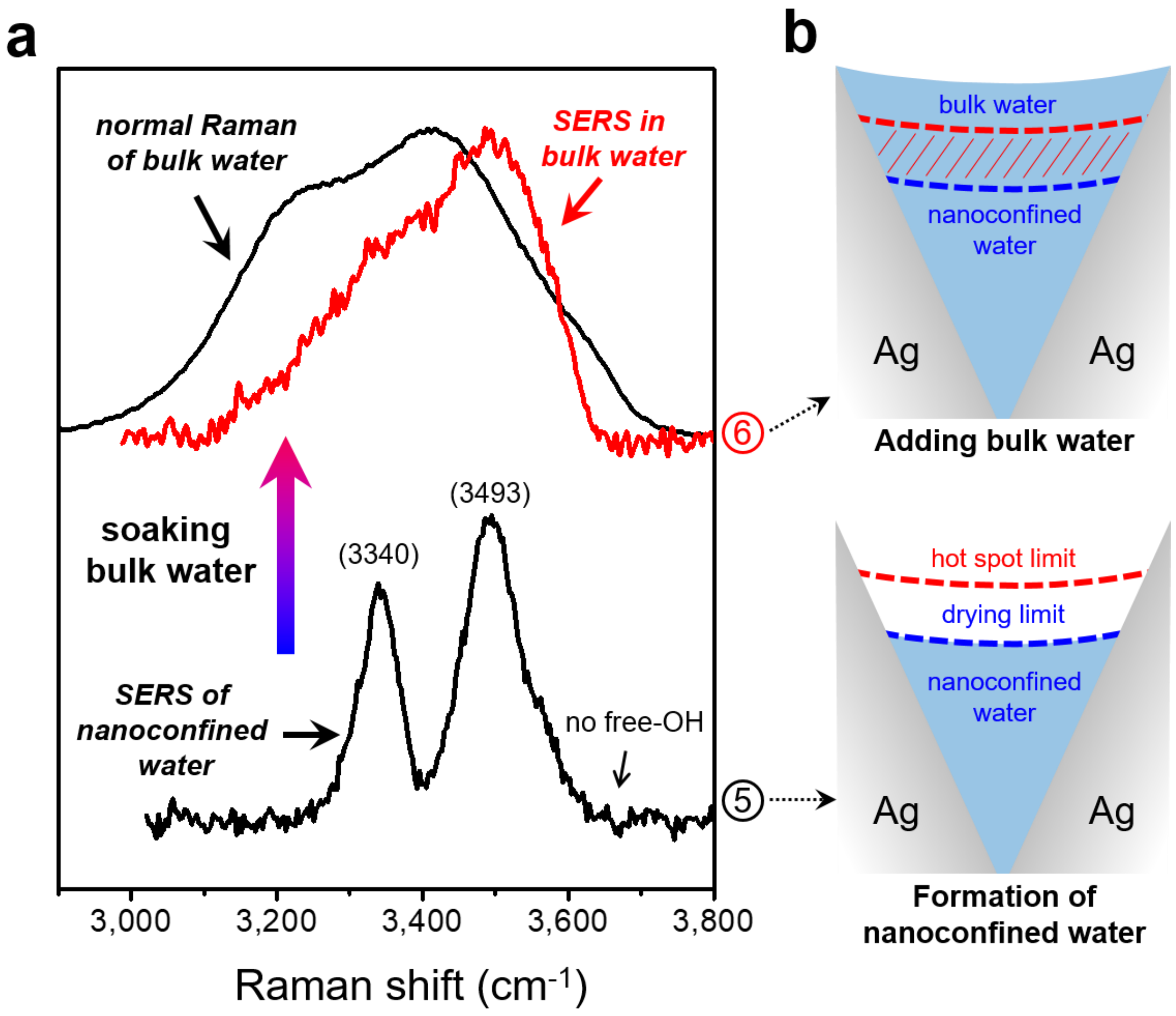

Supplementary Figure 1 | Contribution of added bulk water. a, Adding bulk water in the system after stage 5 contributes to the extra SERS signal that comes from the further filled hot-spot region (red hatched area in **b**), showing the broadened SERS spectrum (red curve in **a**) while still keeping the ice-VII feature.

2. Correlated decrease of the peak ratio between low and high frequency region

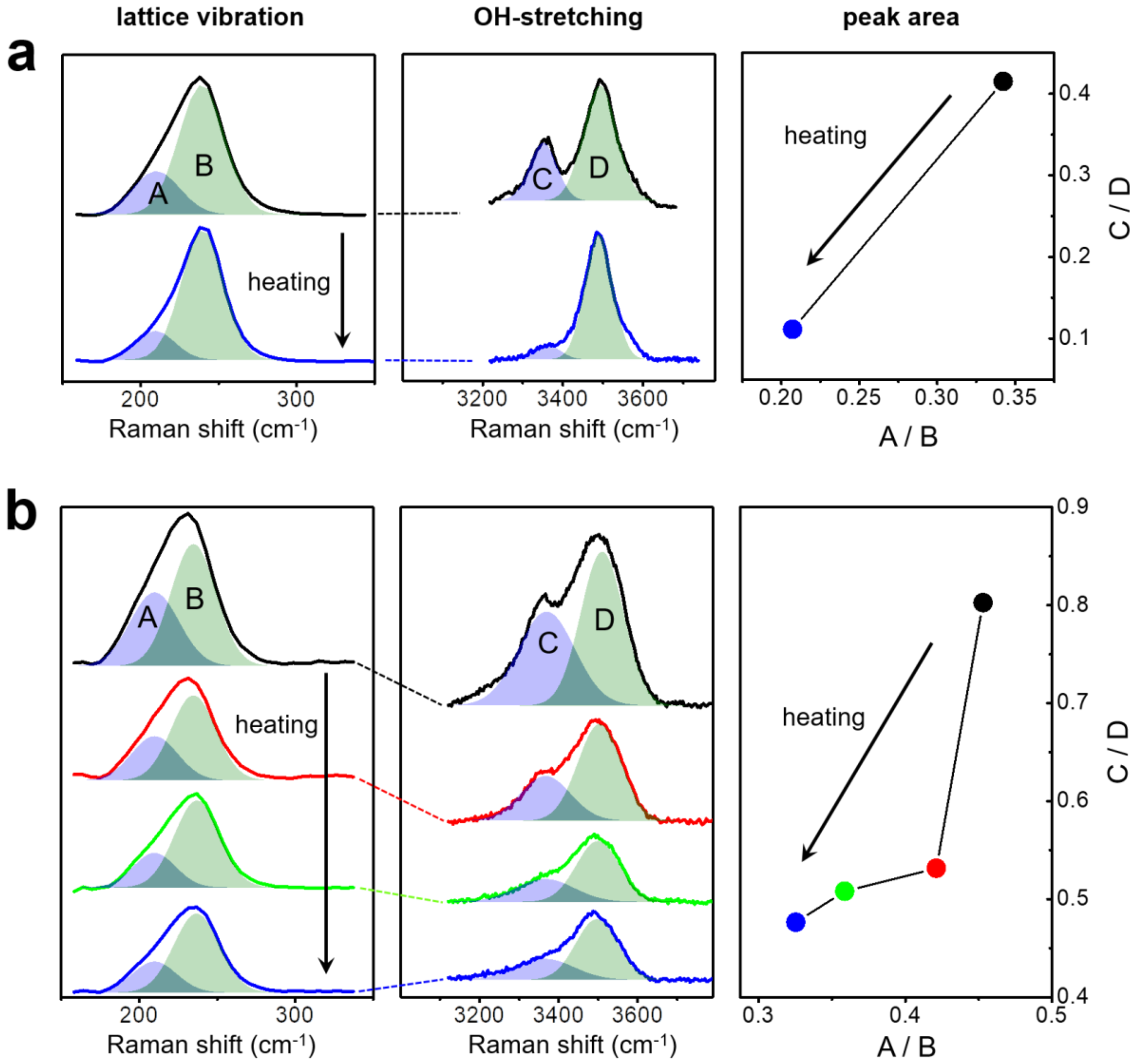

Supplementary Figure 2 | Correlated decrease of the peak ratio between low (A/B) and high (C/D) frequency region is observed during heating experiment (120 °C, 20 min.). **a,** The results are obtained in a separate experiment and are similar to those presented in Fig. 2c in the main text for ultra-purified nanoparticle solution. **b,** The data show the heat-induced change of SERS spectrum of the nanomeniscus produced from less purified nanoparticle solution, which is characterized by the broadened OH stretching bands as is also shown in Fig. 1d in the main text.

3. Raman data of DDAA in most ice phases.

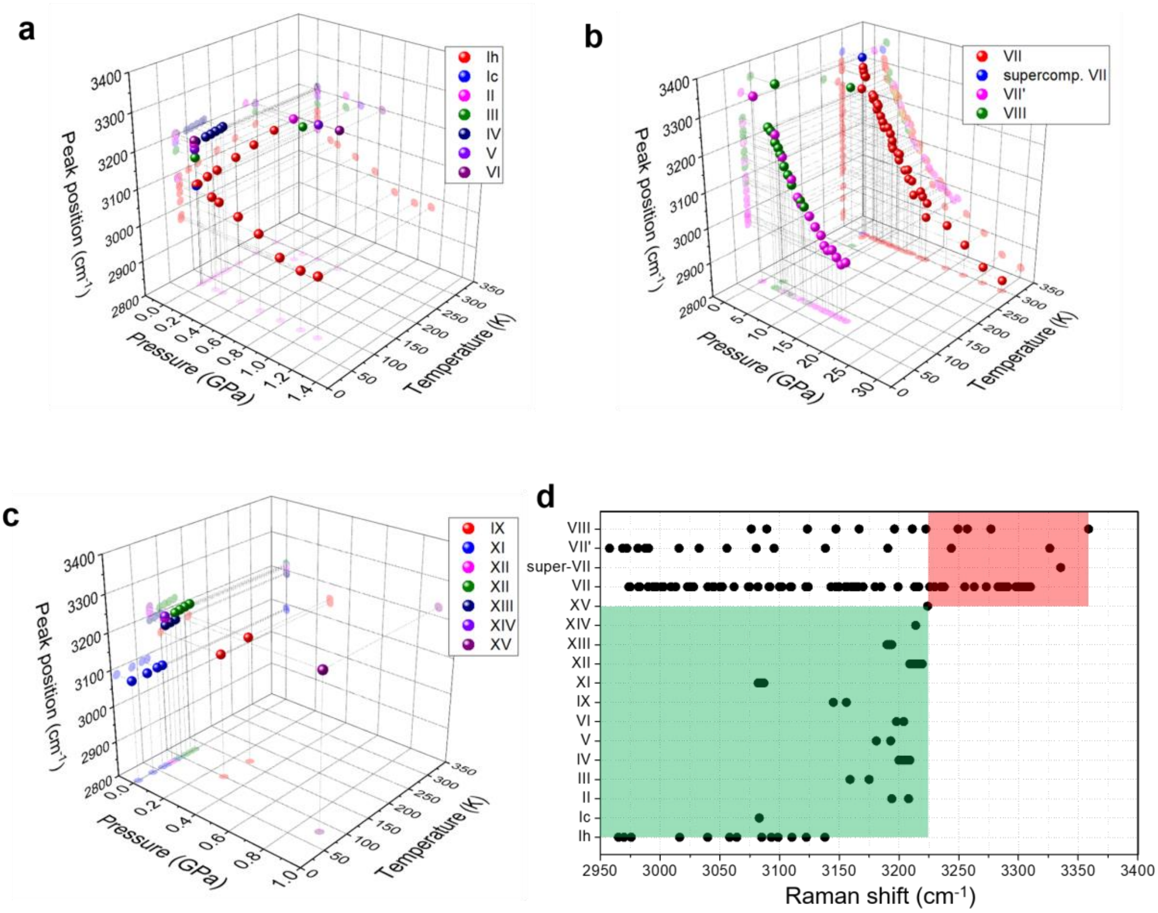

Supplementary Figure 3 | Raman peaks of DDAA in most ice phases versus temperature and pressure. a and **c** show the positions of ice-Ih[2–5], Ic[2], II[2,3], III[2,3], IV[6], V[2,3], VI[3,7], IX[3,8], X[9], XI[4], XII[10], XIII[11], XIV[10] and XV[12], indicating that all the peaks are located below 3225 cm^{-1}, whereas **b** presents the positions of ice-VII[3,7,13–15], ice-VII from supercompressed water[16], VII'[5] and VIII[3,17,18], showing the available peaks above 3250 cm^{-1} at low pressure (clearly visible in **d** as the green- and pink-coloured areas). Notice that the ultra-high pressure X phase has no OH-stretching band.

4. Phase-field diagram of ice-VI and VII.

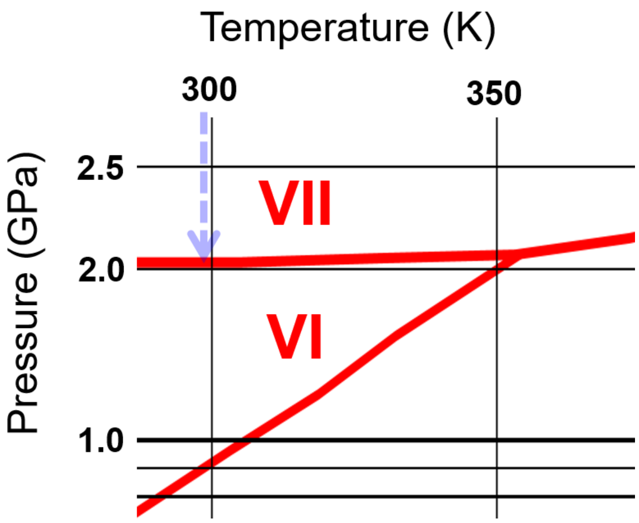

Supplementary Figure 4 | Enlarged phase-field diagram of ice-VI and VII. Blue-dashed arrow shows that the minimum pressure of the ice-VII fields is ~2.1 GPa at 300K.

5. Comparison of amorphous water nanocluster and our nanoconfined water.

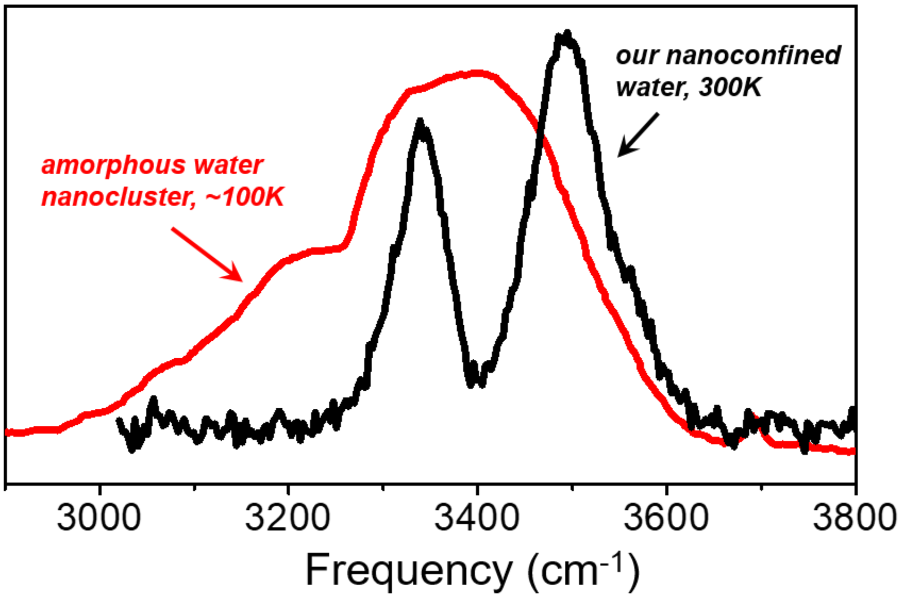

Supplementary Figure 5 | Comparison of bandwidths between water nanocluster and our nanoconfined water. Compared to the infrared spectrum of isolated water nanoclusters (red curve, 48 molecules for 1.5 nm size)[19,20], water nanomeniscus exhibits the significantly narrow bandwidth, verifying the crystalline structure, not the amorphous phase.

6. Change of main phonon band in silicon substrate.

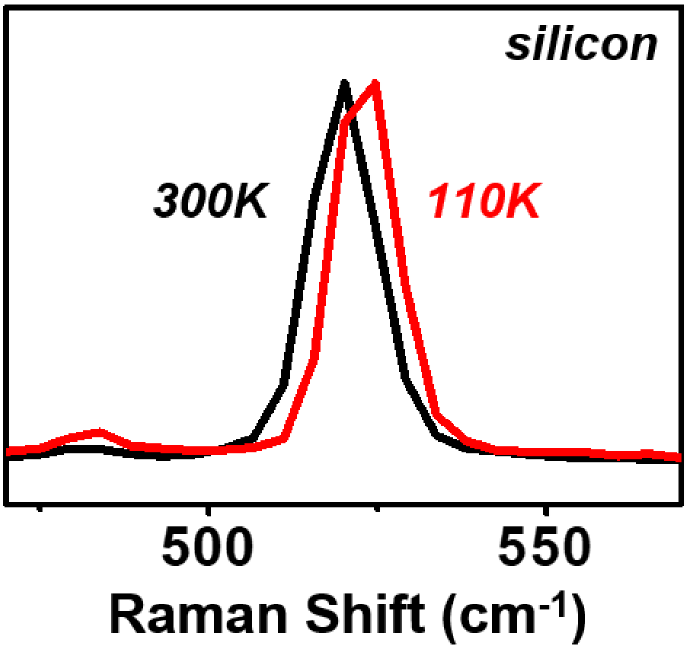

Supplementary Figure 6 | Low temperature change of main phonon band in silicon substrate on which samples are loaded[21]**.**

7. Fabrication and characterization of Au@Ag and Pt@Ag nanoparticle.

We have not used the pure gold or platinum nanoparticle since the plasmonic property (i.e. SERS signal enhancement) of gold or platinum is significantly lower than that of silver nanoparticle, not enough to get reliable SERS of water nanomeniscus, especially at the shorter wavelength region where the Raman cross section of water is increased intrinsically (we used 488 and 532 nm laser line as excitation). Recently, it has been a well established technology to deposit ultrathin gold (or platinum) shell on the core silver nanoparticle, providing silver-dominant plasmonic property along with the gold (or platinum) surface character[22,23].

For gold coating, we followed the recently developed "galvanic replacement-free deposition of gold on silver" method[22], while slightly modifying for our spherical Au@Ag nanoparticle. Briefly, PVP solution (2 mL, 1 mM) was mixed with ascorbic acid (0.5 mL, 10 mM), NaOH solution (0.1 mL, 200 mM) and silver nanoparticle solution (monodispersed or polydispersed, 0.01 mL, ~5 mg/mL) under magnetic stirring. Next, $HAuCl_4$ solution (0.4 mL, 0.1 mM) was titrated into the mixture at a rate of 0.02 mL/min. 10 minutes later, the product was centrifuged at 14,000 rpm and washed with DI water more than five times prior to SERS and TEM characterization (Fig. S6a – d).

For platinum coating[23], Ag nanoparticle solution (~0.3 mg/mL, 3 mL) was initially stabilized with 50 mg of PVP under continuous mixing. Next, reducing agent composed of 100 mg of ascorbic acid (in 3 ml of DI water) and aqueous NaOH solution (600 μL, 1.25 M) was added to the reaction vessel. Pt precursor solution (12 mg of K_2PtCl_4 (99.99%, Sigma-Aldrich) in 16 ml of DI water) was slowly injected at a rate of 4 ml/h for 4 h. Every 2 h, 50 μl of an aqueous 1.25 M NaOH solution was added to the solution. The nanoparticles were purified via centrifugation at 14,000 rpm. with DI water more than five times prior to SERS and TEM characterization. (Fig. S6e – h).

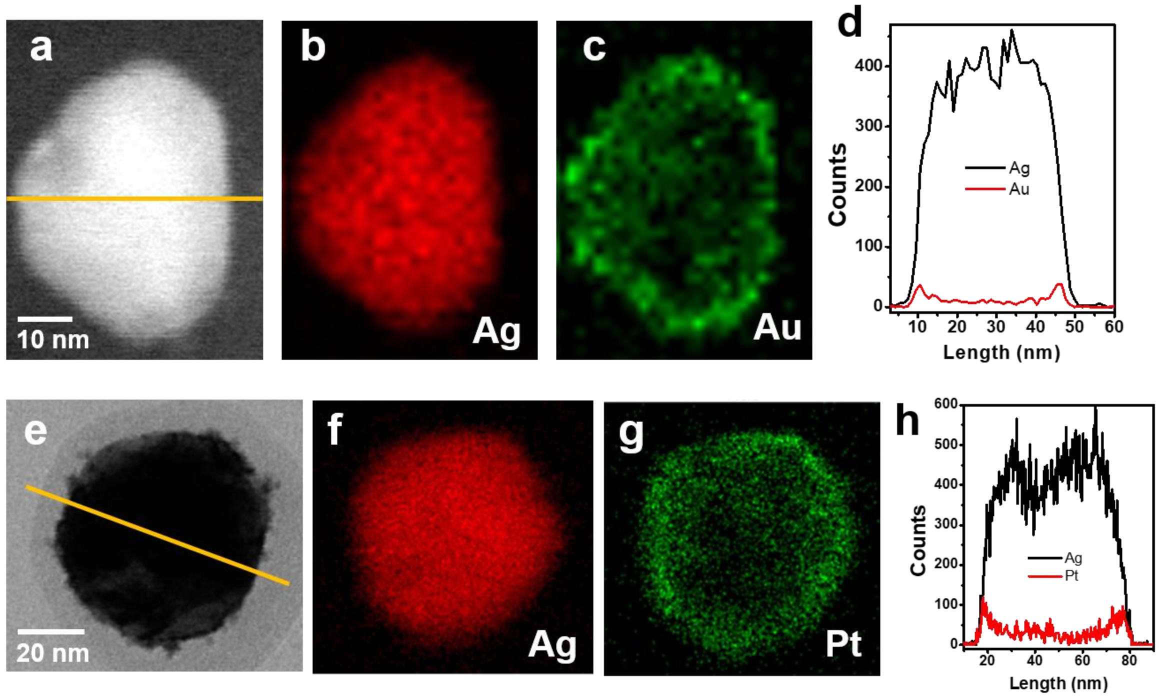

Supplementary Figure 7 | Characterization of Au@Ag and Pt@Ag nanoparticle. a and **e**, TEM images of single Au@Ag and Pt@Ag nanoparticle. EDS elemental maps of Ag (**b, f**), Au (**c**) and Pt (**g**). **d** and **h,** EDS elemental line scan taken along the yellow line in **a** and **e,** respectively.

8. Isocyanide-SERS verification of Au (Pt) surface character in Au@Ag (Pt@Ag).

Since isocyanide group is well known to be sensitively responding to the surface metal characteristics via a spectroscopic shift of the NC stretching band, we assembled the isocyanide SERS probe (1,4-phenylenediisocyanide) on the Au@Ag or Pt@Ag nanoaggregates. Figure S7 clearly shows that the NC stretching band of 1,4-PDI from Au@Ag or Pt@Ag nanoparticle aggregates exhibits the peak position reflecting the Au or Pt surface characters, not Ag one, which is also supported by the previous reports[24–26], confirming the gold (platinum) surface characters of Au@Ag (Pt@Ag) nanoparticle.

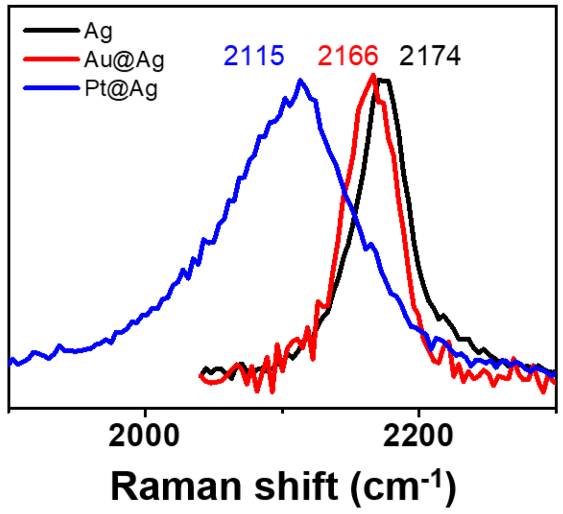

Supplementary Figure 8 | NC stretching bands in SERS of 1,4-phenylenediisocyanide assembled on Ag, Au@Ag and Pt@Ag nanoparticle aggregates.

9. Fabrication of three kinds of OH-SAM@Ag and SERS dependency.

Hydrophilic OH surface was rendered by thiol-based self-assembled monolayer (SAM) molecules: 2-mercpatoethanol (Aldrich, 99%), 3-mercapto-1-propanol (Aldrich, 95%) and 6-mercapto-1-hexanol (Aldrich, 97%), for varying the length of alkyl chain (C2, C3, C6). The Ag nanoparticles were coated by these OH-SAM molecules by adding 10 μl ethanolic solutions to 1ml Ag colloids with enough amount of PVP stabilizer, to prevent from aggregation in the solution state. After incubating the mixture for 10 minutes at room temperature, solutions were washed more than 5 times with DI water for SERS measurement of water nanomeniscus.

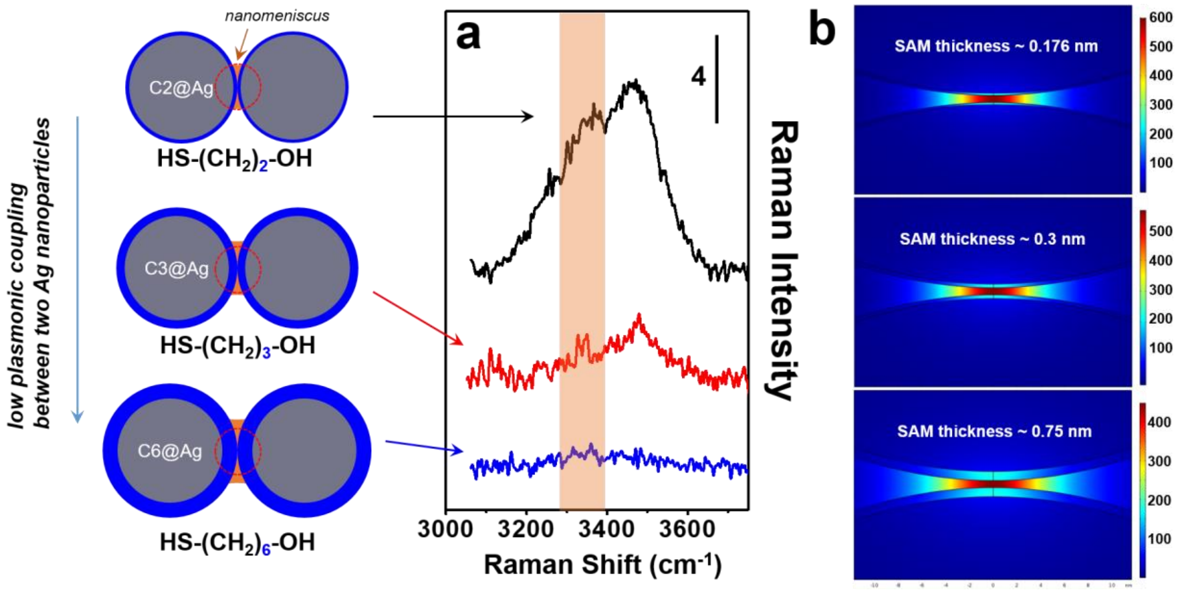

Supplementary Figure 9 | Plasmonic coupling (SERS hot spot) between adjacent silver nanoparticles. **a**, Effective plasmonic coupling is decreased with the increasing alkyl chain length of the OH-SAM molecules, so that SERS of water nanomeniscus formed at the crevices becomes difficult to be observed with the increasing number of alkyl group. (SAM thickness of 0.175 nm, 0.3 nm and 0.75 nm for C2, C3 and C6 SAM, respectively[27,28]. Scale bar in Raman spectra indicates the Raman counts at the condition of 1 mW at 532 nm, 1 second integration time and 90 accumulations.) **b**, FEM simulation results show that the electromagnetic field intensity in the region where nanomeniscus forms decreases significantly with the increasing shell (OH-SAM) thickness of the particle (0.175 nm, 0.3 nm and 0.75 nm). The refractive index value for the OH-SAM was provided by the result in the previous report[29]. Scale bar denotes the intensity of scattered electromagnetic field |E| near the crevice.

Now, let us discuss the plausibility of SERS of the water nanomeniscus: OH-SAM@Ag vs SHINER (Shell-isolated Nanoparticle-enhanced Raman Spectroscopy) particle.

At first, we expected the appearance of SERS of the water nanomeniscus especially at the silica surface since it has the surface characteristic of the natural sands. However, we observed experimentally in Figure S9 (theoretically in Fig. S9b) that the SERS of the water nanomeniscus critically depends on the length of alkyl group of OH-SAM, indicating that if the thickness of the SAM is over 1 nm (C6), SERS of the water nanomeniscus is hardly detectable at the crevices of the particle aggregates. Considering that the minimum thickness of silica shell in SHINER nanoparticle is 1 nm up to now[30,31], SERS of the water nanomeniscus was not observed in the aggregates of SHINER nanoparticle as expected.

10. Fabrication of monodispersed silver nanoparticles.

For monodispersed Ag nanoparticles, we followed the protocol of using mixture of glycerol and water, developed by Schlücker's group[32]. Briefly, a 50 mL glycerol–water mixture (40 vol% glycerol) was stirred in a 100 mL flask and heated up to 95 ˚C. After a few minutes later, 9 mg silver nitrate along with 1 mL sodium citrate (3%) were added to the solvent, followed by stirring for 1 hour at 95 ˚C. Then, using this particle as seed and combining with glycerol, PVP and ascorbic acid, we synthesized monodisperse Ag nanoparticles of 43 nm, 70 nm and 100 nm sizes, which are confirmed by narrow bandwidth and maximum peak position in the extinction spectrum (Fig. S10).

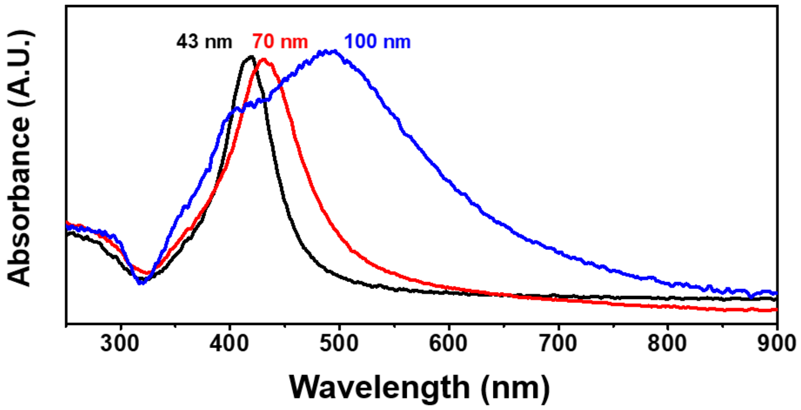

Supplementary Figure 10 | UV-VIS Absorbance of monodispersed Ag nanoparticles. Diameter is denoted near each spectrum.

11. Fabrication of polydispersed silver nanoparticle.

For polydispersed silver nanoparticle, we used the modified polyol synthesis[23] which is generally used for silver nanocube and very sensitive to the environmental conditions. We slightly changed the reaction conditions such as heating temperature or reagent concentration. Polydispersity of the sample is supported by the large bandwidth in absorbance spectrum (Fig. S10) along with field-emission scanning electron microscopic (FESEM) investigation, showing the coexistence of various kinds of rod, wire and polyhedral shape of nanoparticles in the dried aggregates (Fig. S11).

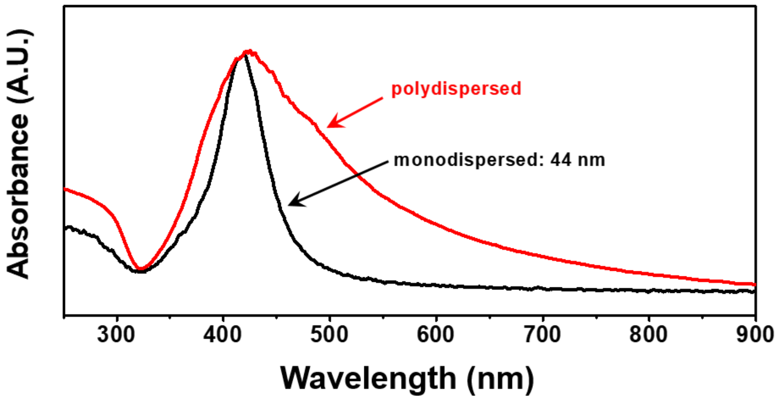

Supplementary Figure 11 | UV-VIS Absorbance of monodispersed (44 nm diameter) and polydispersed Ag nanoparticle solution.

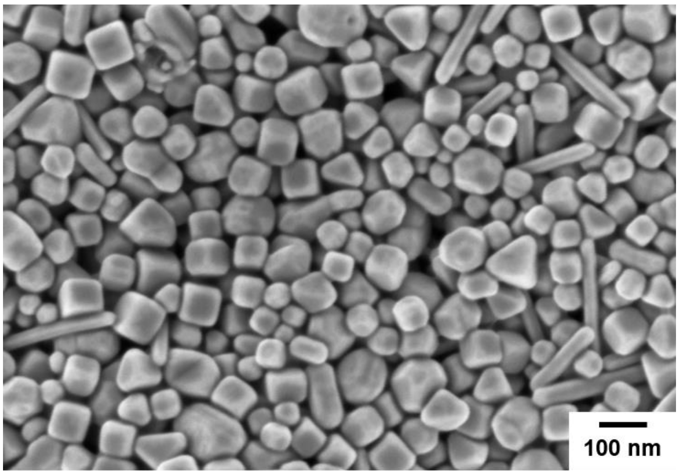

Supplementary Figure 12 | FE-SEM image of dried polydispersed Ag nanoparticles.

12. Correlated Low and high frequency bands in various systems of our experiments.

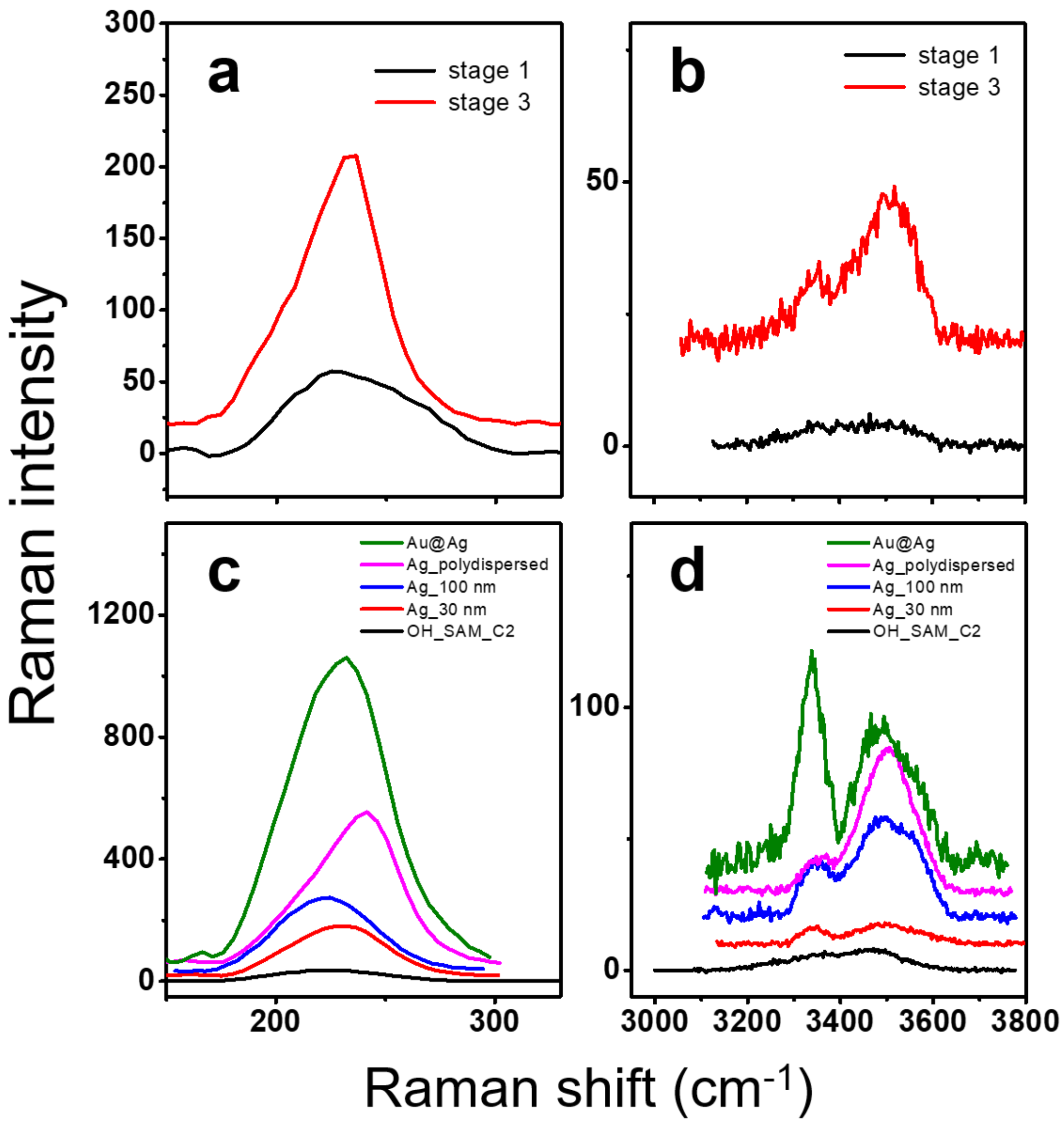

Supplementary Figure 13. Correlated behavior between low and high frequency bands observed in the washing stages (**a** and **b**) and in the various kinds of nanoparticles (**c** and **d**).

13. SERS of water nanomeniscus in different forms of silver substrate.

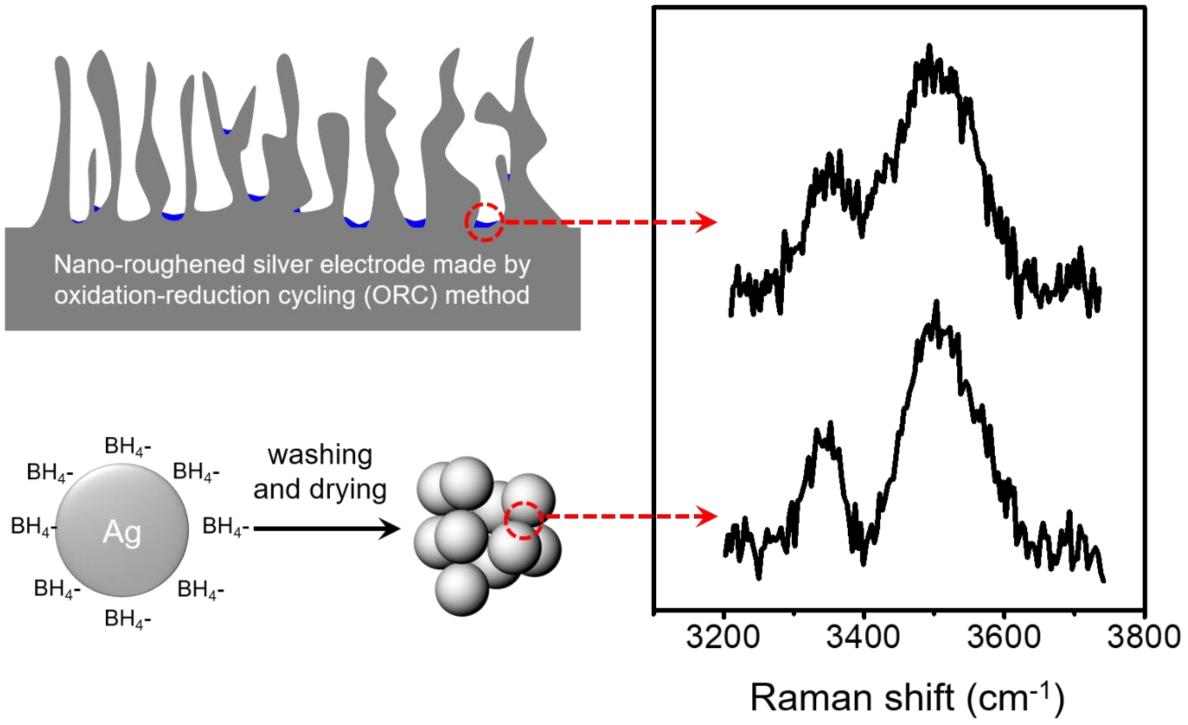

Supplementary Figure 14 | SERS of water nanomeniscus in silver substrate made by different methods. The nano-roughened silver electrode produced by the conventional electrochemical-ORC (oxidation reduction cycle) method[33] as well as the dried nanoaggregates made by the $NaBH_4$-reduced silver nanoparticle[34] show a very similar SERS feature of the nanoconfined water in ambient condition that is presented in the main text (i.e., that produced by the sodium citrate-reduced silver nanoaggregates). The results confirm that the observed SERS peak of the nanoconfined water meniscus is not due to other silver-water or other impurity-water complex compounds that can be produced during experiments.

14. Ultra-sonication effect on the SERS spectrum.

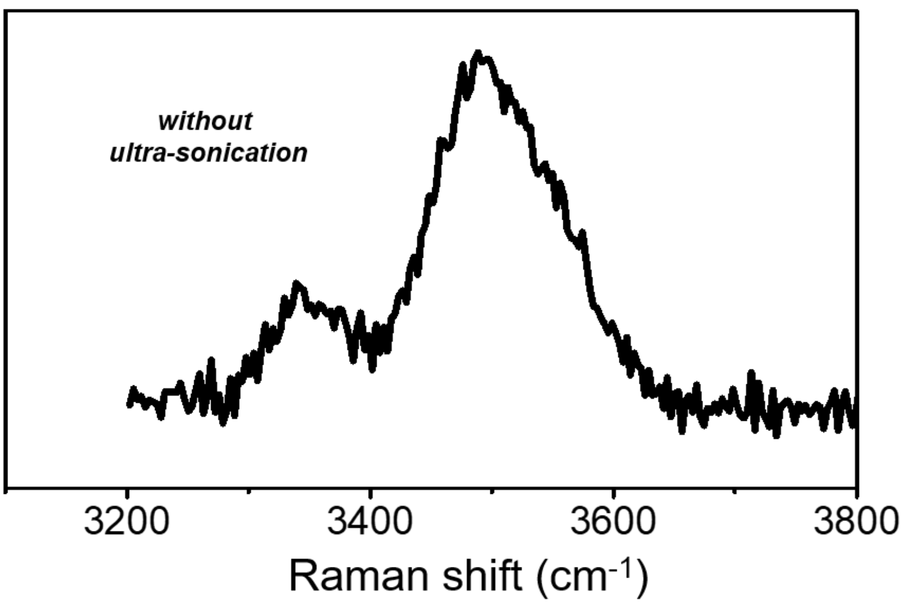

Supplementary Figure 15 | Ultra-sonication effect on the SERS spectrum. Silver nanoparticle is known to be sensitive to the external stimuli. To examine whether the ultra-sonication procedure leads to formation of other kinds of silver-water complex compound (besides the nanoconfined water meniscus), we diluted the solution 15 times only with the distilled water without any ultra-sonication treatment in the sample preparation stage. The dried aggregates prepared by this method also exhibit the similar spectral features of ice-VII. The results show that the SERS peak of water nanomeniscus is not associated with any sonication-induced products that may be formed during experiments.

Supplementary Notes 1. Estimates of SERS enhancement factor and near-surface pressure.

(i) SERS enhancement factor

The signal enhancement factor (EF) is defined as, $\mathrm{EF} = \frac{I_{\mathrm{SERS}}}{N_{\mathrm{SERS}}} / \frac{I_{\mathrm{bulk}}}{N_{\mathrm{bulk}}}$, where I and N denote the Raman scattering intensity and number of probed molecules, respectively. We irradiated with 20-mW laser light (532 nm) for bulk-water Raman measurement due to the very low optical Raman cross-section, while 1mW laser was used for the nanomeniscus SERS measurement. Since we obtained the same Raman signal intensity with each laser power, the ratio of $\mathrm{I_{SERS}/I_{bulk}}$ is ~20. For bulk-water measurement, the sampling volume is assumed as a cylinder geometry with ~2 μm diameter with 20 μm height and the water density of 1 g/ml, giving the N_{bulk} value as $\sim 2.1 \times 10^{12}$. The volume of single hot-spot area between two silver nanospheres is derived by a simple geometrical calculation using the FEM method, which provides N_{SERS} ~ 775 water molecules in the single hot spot region. In the randomly dried nanoaggregates, we estimate the maximum EF value (< 5.42×10^{7}), from the hot spot numbers (< 1000 in the single laser spot of ~2 μm), although it is difficult to count the exact number by analyzing the TEM (transmission electron microscope) or FE-SEM (field emission scanning electron microscope) images of the samples.

(ii) Pressure near the surface

We estimate the pressure exerted on the single water molecule situated near the silver surface. The force between a water dipole $\mathrm{P} = \mathrm{q} \times \delta_0$ (=1.8546 D) and its image on the metal is calculated by the image-dipole equation[1], $\mathrm{F(d)} \approx \frac{3\mathrm{P}^2}{4\pi\epsilon_0(2\mathrm{d})^4}$, where d is the distance between the dipole and its image. If we assume that the cross-sectional area of a single water molecule

on the silver surface is ~ $(0.3\ nm)^2$ and the distance between a confined water molecule and its image has the molecular size of single water (~ 0.3 nm), the pressure between them is calculated qualitatively as ~1.37 GPa. For comparison, if the distance between a surface adsorbed water (in direct contact with the silver surface) and its image is on the order of atomic diameter of oxygen (~ 0.12 nm), the pressure is obtained as ~3 GPa, supporting that the surface water molecules are more strongly bound than the confined ones, as also manifested by the invariant DA peak of the nanoconfined water during heating of the system.

References

1. Teodorescu, C. M. Image molecular dipoles in surface enhanced Raman scattering. *Phys. Chem. Chem. Phys.* **17,** 21302–21314 (2015).

2. Taylor, M. & Whalley, E. Raman spectra of ices Ih, Ic, II, III, and V. *J. Chem. Phys.* **40,** 1660 (1964).

3. Wilkinson, G. R. The Raman spectra of ice (Ih, II, III, V, VI and IX) as functions of pressure and temperature. *J. Phys. C Solid state Phys.* **17,** 5833–5850 (1984).

4. Furić, K. & Volovšek, V. Water ice at low temperatures and pressures: New Raman results. *J. Mol. Struct.* **976,** 174–180 (2010).

5. Yoshimura, Y., Stewart, S. T., Somayazulu, M., Mao, H. K. & Hemley, R. J. Convergent raman features in high density amorphous ice, ice VII, and ice VIII under pressure. *J. Phys. Chem. B* **115,** 3756–3760 (2011).

6. Salzmann, C. G., Kohl, I., Loerting, T., Mayer, E. & Hallbrucker, A. Raman spectroscopic study on hydrogen bonding in recovered ice IV. *J. Phys. Chem. B* **107,** 2802–2807 (2003).

7. Marckmann, J. P. & Whalley, E. Vibrational spectra of the ices. Raman spectra of ice VI and Ice VII. *J. Chem. Phys.* **41,** 1450–1453 (1964).

8. Minčeva-Śukarova, B. Shermann, W. F. & Wilkinson, G. R. A high pressure spectroscopic study on the ice III - ice IX. Disordered - ordered transition. *J. Mol. Struct.* **115,** 137–140 (1984).

9. Goncharov, A. F., Struzhkin, V. V., Mao, H. & Hemley, R. J. Raman spectroscopy of dense H2O and the transition to symmetric hydrogen bonds. *Phys. Rev. Lett.* **83,** 1998–2001 (1999).

10. Salzmann, C. G., Hallbrucker, A., Finney, J. L. & Mayer, E. Raman spectroscopic features of hydrogen-ordering in ice XII. *Chem. Phys. Lett.* **429,** 469–473 (2006).

11. Salzmann, C. G., Hallbrucker, A., Finney, J. L. & Mayer, E. Raman spectroscopic study of hydrogen ordered ice XIII and of its reversible phase transition to disordered ice V. *Phys. Chem. Chem. Phys.* **8,** 3088–3093 (2006).

12. Whale, T. F., Clark, S. J., Finney, J. L. & Salzmann, C. G. DFT-assisted interpretation of the Raman spectra of hydrogen-ordered ice XV. *J. Raman Spectrosc.* **44,** 290–298 (2013).

13. Walrafen, G. E. *et al.* Raman and x-ray investigations of ice VII to 36.0 GPa. *J. Chem. Phys.* **77,** 2166–2174 (1982).

14. Zha, C. *et al.* New Raman measurements for H2O ice VII in the range of 300 cm-1 to 4000 cm-1 at pressures up to 120 GPa. *J. Chem. Phys.* **145,** 124315 (2016).

15. Pruzan, P., Chervin, J. C. & Gauthier, M. Raman spectroscopy investigation of ice VII and deuterated ice VII to 40 GPa. Disorder in ice VII. *Europhys. Lett.* **13,** 81–87 (1990).

16. Lee, G. W., Evans, W. J. & Yoo, C. S. Crystallization of water in a dynamic diamond-anvil cell: Evidence for ice VII-like local order in supercompressed water. *Phys. Rev. B - Condens. Matter Mater. Phys.* **74,** 1–6 (2006).

17. Hirsch, K. R. & Holzapfel, W. B. Effect of high pressure on the Raman spectra of ice VIII and evidence for ice X. *J. Chem. Phys.* **84,** 2771–2775 (1986).

18. Wong, P. T. T. & Whalley, E. Raman spectrum of ice VIII. *J. Chem. Phys.* **64,** 2359 (1976).

19. Pradzynski, C. C., Forck, R. M., Zeuch, T., Slavíček, P. & Buck, U. A fully size-resolved perspective on the crystallization of water clusters. *Science* **337,** 1529–1532 (2012).

20. Buch, V. & Devlin, J. P. in *Water in confining geometries* 447 (Springer-Verlag Berlin Heidelberg, 2003). doi:10.1007/978-3-662-05231-0

21. Hart, T., Aggarwal, R. & Lax, B. Temperature Dependence of Raman Scattering in Silicon. *Phys. Rev. B* **1,** 638–642 (1970).

22. Yang, Y., Liu, J., Fu, Z. & Qin, D. Galvanic Replacement-Free Deposition of Au on Ag for Core−Shell.pdf. *J. Am. Chem. Soc.* 1–12 (2014).

23. Aslam, U., Chavez, S. & Linic, S. Controlling energy flow in multimetallic nanostructures for plasmonic catalysis. *Nat. Nanotechnol.* **12,** 1000–1005 (2017).

24. Kim, H. S. *et al.* Adsorption characteristics of 1,4-phenylene diisocyanide on gold nanoparticles: Infrared and Raman spectroscopy study. *Langmuir* **19,** 6701–6710 (2003).

25. Han, H. S., Han, S. W., Joo, S. W. & Kim, K. Adsorption of 1 , 4-Phenylene Diisocyanide on Silver Investigated by Infrared and Raman Spectroscopy. 6868–6874 (1999).

26. Shin, D. All Platinum-Made Gap Mode Nanostructure for Surface Enhanced Raman Spectroscopy. *J. Phys. Chem. C* **120,** 13701–13705 (2016).

27. Love, J. C., Estroff, L. A., Kriebel, J. K., Nuzzo, R. G. & Whitesides, G. M. *Self-assembled monolayers of thiolates on metals as a form of nanotechnology*. *Chemical Reviews* **105,** (2005).

28. Porter, M. D., Bright, T. B., Allara, D. L. & Chidsey, C. E. Spontaneously Organized Molecular Assemblies. 4. Structural Characterization of n-Alkyl Thiol Monolayers on Gold by Optical Ellipsometry, Infrared Spectroscopy, and Electrochemistry. *J. Am. Chem. Soc.* **109,** 3559–3568 (1987).

29. Rampi, M. A., Schueller, O. J. A. & Whitesides, G. M. Alkanethiol self-assembled monolayers as the dielectric of capacitors with nanoscale thickness Alkanethiol self-assembled monolayers as the dielectric of capacitors with nanoscale thickness. *Appl. Phys. Lett.* **72,** 1781–1783 (1998).

30. Li, J. F. *et al.* Shell-Isolated Nanoparticle-Enhanced Raman Spectroscopy (SHINERS). *Nature* **464,** 392–395 (2010).

31. Li, C. Y. *et al.* “Smart” Ag Nanostructures for Plasmon-Enhanced Spectroscopies. *J. Am. Chem. Soc.* **137,** 13784–13787 (2015).

32. Steinigeweg, D. & Schlücker, S. Monodispersity and size control in the synthesis of 20-100 nm quasi-spherical silver nanoparticles by citrate and ascorbic acid reduction in glycerol-water mixtures. *Chem. Commun.* **48,** 8682–8684 (2012).

33. Jeanmaire, D. L. & Van Duyne, R. P. Surface Raman spectroelectrochemistry: Part I.

Heterocyclic, aromatic, and aliphatic amines adsorbed on the anodized silver electrode. *J. Electroanal. Chem.* **84,** 1–20 (1977).

34. Lee, P. C. & Meisel, D. Adsorption and surface-enhanced Raman of dyes on silver and gold sols. *J. Phys. Chem.* **86,** 3391–3395 (1982).
